# Conservation and loss of ribosomal RNA gene sites in diploid and polyploid *Fragaria *(Rosaceae)

**DOI:** 10.1186/1471-2229-11-157

**Published:** 2011-11-10

**Authors:** Bo Liu, Thomas M Davis

**Affiliations:** 1 Department of Biological Sciences, University of New Hampshire, Durham, NH 03824, USA

## Abstract

**Background:**

The genus *Fragaria *comprises species at ploidy levels ranging from diploid (2*n *= 2*x *= 14) to decaploid (2*n *= 10*x *= 70). Fluorescence *in situ *hybridization with 5S and 25S rDNA probes was performed to gather cytogenetic information that illuminates genomic divergence among different taxa at multiple ploidy levels, as well as to explore the evolution of ribosomal RNA genes during polyploidization in *Fragaria*.

**Results:**

Root tip cells of diploid taxa were typified by two 5S and six 25S rDNA hybridization signals of varying intensities, providing a baseline for comparisons within the genus. In three exceptional diploid genotypes, *F. nilgerrensis *(CFRA 1358 and CFRA 1825) and *F. vesca *'Yellow Wonder', two 5S but only four 25S rDNA sites were found but with differing site losses. The numbers of 5S and 25S rDNA signals, respectively were three and nine in a triploid *F*. ×*bifera *accession, and were four and twelve in three tetraploids, thus occurring in proportional 1.5× and 2× multiples of the typical diploid pattern. In hexaploid *F*. *moschata*, a proportional multiple of six 5S rDNA sites was observed, but the number of 25S rDNA sites was one or two less than the proportionate prediction of eighteen. This apparent tendency toward rDNA site loss at higher ploidy was markedly expanded in octoploids, which displayed only two 5S and ten 25S rDNA sites. In the two decaploids examined, the numbers of 5S and 25S rDNA signals, respectively, were four and fifteen in *F. virginiana *subsp. *platypetala*, and six and twelve in *F. iturupensis*.

**Conclusions:**

Among diploid *Fragaria *species, a general consistency of rDNA site numbers implies conserved genomic organization, but highly variable 25S signal sizes and intensities and two instances of site loss suggest concurrent high dynamics of rDNA copy numbers among both homologs and non-homologs. General conservation of rDNA site numbers in lower ploidy, but marked site number reductions at higher ploidy levels, suggest complex evolution of rDNA sites during polyploidization and/or independent evolutionary pathways for 6*x *versus higher ploidy strawberries. Site number comparisons suggest common genomic composition among natural octoploids, and independent origins of the two divergent decaploid accessions.

## Background

The strawberry genus *Fragaria *belongs to the Rosaceae, an economically important plant family, and includes about 24 species distributed mostly throughout the temperate zone of Europe, Asia, North and South America [1,2]. Historically, a basic chromosome number of seven (*x *= 7) and the existence of multiple levels of ploidy, ranging from diploid to octoploid, in this genus had been documented by 1926 [3,4]. Only recently, a naturally occurring decaploid cytotype (2*n *= 10*x *= 70) was revealed through chromosome counting and flow cytometry [5] in the geographically isolated species *F. iturupensis*, which had initially been described as an octoploid (2*n *= 8*x *= 56) [6]. In addition, an accession (CFRA 110) of *F*. *virginiana *subsp. *platypetala *has been found by chromosome counting to be decaploid [7]. Currently, twelve diploid (2*n *= 2*x *= 14), five tetraploid (2*n *= 4*x *= 28), a single hexaploid (2*n *= 6*x *= 42) *F. moschata *, three octoploid (2*n *= 8*x *= 56) species, including the main cultivated species *F*. ×*ananassa *and its immediate octoploid ancestors: *F. chiloensis *and *F. virginiana *[8], and a decaploid (2*n *= 10*x *= 70) *F. iturupensis *are recognized [1,5,9,10]. In addition, variable- and odd-ploidy have been recognized in two hybrid taxa: pentaploid/hexaploid/enneaploid (2*n *= 5*x*/6*x*/9*x *= 35/42/63) *F*. ×*bringhurstii *[11] and diploid/triploid (2*n *= 2*x*/3*x *= 14/21) *F*. ×*bifera *[12]. The higher level (> 4*x*) polyploids are all considered to have at least partially allopolyploid genome compositions; however, classifications of origins as auto- or allo-ploidy have not been resolved at the tetraploid level [13].

Non-isotopic *in situ *hybridization (ISH) was introduced in plants in 1985 [14], and fluorescence-based techniques (FISH) have subsequently become routine methods for physical mapping of repetitive DNA sequences and multicopy gene families [15] and other DNA sequences onto chromosomes [16], as well as for the identification of individual chromosomes (e.g., [17]). Ribosomal RNA (rRNA) genes have been the most widely targeted probe sites due to their high copy numbers, specific chromosomal positions and the high degree of sequence conservation among different plant groups [18,19]. In eukaryotes, including higher plants, nuclear 18S, 5.8S and 25/28S rRNAs (25S in plants; 28S in mammals) [20,21] result from processing of a 45S transcript encoded by rDNA repeated units clustered at particular chromosomal sites. The 5S rRNA results from transcription of distinct gene clusters located in different chromosomal sites [22].

Previous reports on cytogenetics in strawberry are mostly limited to chromosome counting, as initiated by Ichijima [3] and Longley [4], and more recently to traditional karyotype analysis [7,23-26]. In the field of modern molecular cytogenetics, in which fluorescence *in situ *hybridization (FISH) techniques play a key role, work on strawberry has been minimal. Feasibility of FISH with rDNA probes was first established by Lim [27] in diploid strawberry, with confirmation by Shulaev et al. [28], but the technique has yet to be extended to the genetically and cytologically more complex genomes of the polyploid *Fragaria *species. As a genus that spans ploidy levels from diploid through decaploid, the genus *Fragaria *offers a previously untapped opportunity for studying the evolutionary changes in chromosomal rDNA arrays that have occurred during polyploidy evolution, and will contribute to ongoing efforts to define the genomic composition(s) of the polyploid strawberry species. Moreover, we anticipate that the study reported here will contribute to further development of comparative molecular cytogenetics in Rosaceae, as strawberry is one of the best-developed model organisms for this family [29].

The objectives of this work were to characterize the genomic distribution of 5S and 25S rDNA arrays in *Fragaria *species, and to assess the changes in rDNA site number that are associated with polyploidization in *Fragaria*.

## Results

Numbers of 5S and 25S rDNA sites visualized by fluorescence *in situ *hybridization in one or more accessions of each studied taxon are shown (Table 1). For each accession, at least five cells with good chromosome spreads and hybridization signals were observed. In all the species including the diploids and polyploids, 5S rDNA sites were all localized on proximal regions of short chromosome arms, and 25S rDNA sites were all localized on terminal chromosomal regions. The site numbers of both rDNA types was consistent within each level of ploidy, with the following exceptions. As detailed below, five diploid cytotypes had less than the number of 25S signals presented by the typical diploid cytotype. The intensity and size pattern of 25S signals displayed variability within the diploids. Also, a difference of 25S site number was observed between the two hexaploid *F. moschata *(CFRA 157 and CFRA 376) genotypes. Finally, the two decaploid taxa differed from each other in both 5S and 25S site numbers.

**Table 1:** Numbers of 5S and 25S rDNA sites in different *Fragaria *spp.

Species	Ploidy	Accession	rDNA site number	
			
			5S	25S
*F. bucharica*	2*x*	CFRA 520	2*	6
*F. daltoniana*	2*x*	CFRA 1685	2*	6
*F. iinumae*	2*x*	CFRA 1850	2*	6
*F. mandshurica*	2*x*	CFRA 1947	2*	6
*F. nilgerrensis*	2*x*	CFRA 1358	2*	4
*F. nilgerrensis*	2*x*	CFRA 1825	2*	4
*F. nipponica*	2*x*	CFRA 1862	2*	5
*F. pentaphylla*	2*x*	GS34	2*	6
*F. vesca *ssp. *americana*	2*x*	CFRA 1948	2 = 1*+ 1	5
*F. vesca *ssp. *americana*	2*x*	'WC6'	2*	6
*F. vesca *ssp. *bracteata*	2*x*	'BC32'	2*	6
*F. vesca *ssp. *bracteata *or *californica*	2*x*	CFRA 1990	2*	6
*F. vesca *ssp. *bracteata *or *californica*	2*x*	'HP6A'	2*	6
*F. vesca *ssp. *vesca*	2*x*	CFRA 438	2*	6
*F. vesca *ssp. *vesca*	2*x*	'NOV 1C'	2*	6
*F. vesca *ssp. *vesca*	2*x*	'Yellow Wonder'	2	4
*F. vesca *ssp. *vesca*	2*x*	'Hawaii 4'	2*	6
*F. viridis*	2*x*	CFRA 333	2*	6
*F. ×bifera*	3*x*	GS104	3*	9
*F. corymbosa*	4*x*	CFRA 1911	4*	12
*F. gracilis*	4*x*	GS31	4*	12
*F. tibetica*	4*x*	GS28	4*	12
*F. moschata*	6*x*	CFRA 157	6 = 5*+1	16
*F. moschata*	6*x*	CFRA 376	6 = 5*+1	17
*F. chiloensis *ssp. *chiloensis *f. *patagonica*	8*x*	CFRA 1100	2	10
*F. chiloensis *ssp. *lucida*	8*x*	CFRA 1691	2	10
*F. chiloensis *ssp. *pacifica*	8*x*	CFRA 48	2	10
*F. virginiana *ssp. *glauca*	8*x*	CFRA 1992	2	10
*F. virginiana *ssp. *glauca*	8*x*	CFRA 370	2	10
*F. virginiana *ssp. *grayana*	8*x*	CFRA 1408	2	10
*F. virginiana *ssp. *virginiana*	8*x*	CFRA 1994	2	10
*F. virginiana *ssp. *platypetala*	10*x*	CFRA 110	4 = 1*+3	15
*F. iturupensis*	10*x*	CFRA 1841	6	12

* chromosomes bearing both 25S and 5S rDNA sites.

Local numbers are shown here if CFRA numbers are not available.

### Genomic distribution of 5S and 25S rDNA sites in diploids

**Figure 1:**
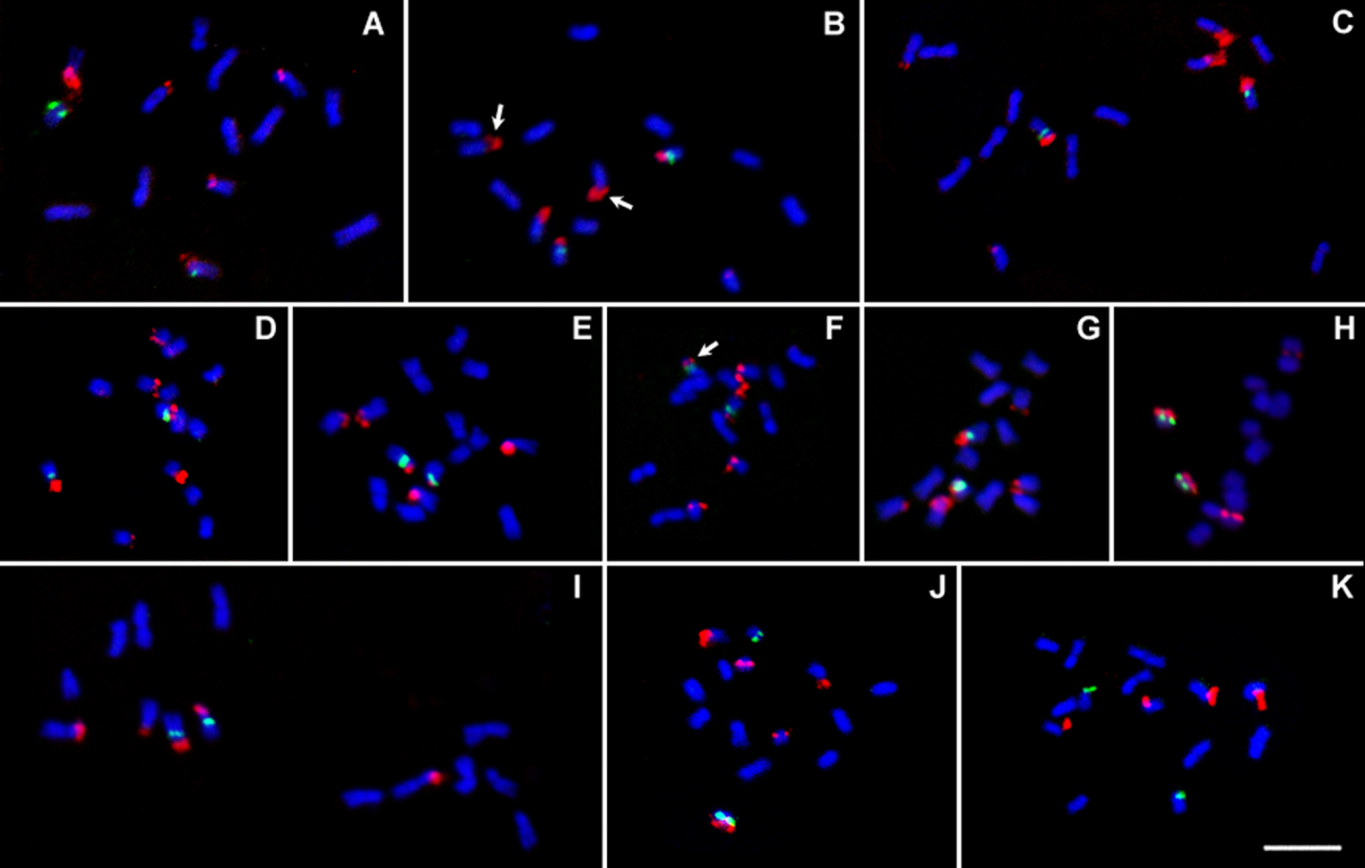
**FISH on diploid *Fragaria *genotypes with 5S (green signals) and 25S (red signals) rDNA probes**. **A: ***F. bucharica *(CFRA 520); **B: ***F. mandshurica *(CFRA 1947); **C: ***F. vesca *subsp. *vesca *'Hawaii 4'; **D: ***F. daltoniana *(CFRA 1685); **E: ***F. pentaphylla *'GS34'; **F: ***F. viridis *(CFRA 333); **G: ***F. iinumae *(CFRA 1850); **H: ***F. nilgerrensis *(CFRA 1358); **I: ***F. nipponica *(CFRA 1862); **J: ***F*. *vesca *subsp. *americana *'Pawtuckaway' (CFRA 1948); **K: ***F. vesca *subsp. *vesca *'Yellow Wonder'. Arrows show locations of satellites that are visible under DAPI counterstain. Bar = 5 μm.

**Figure 2:**
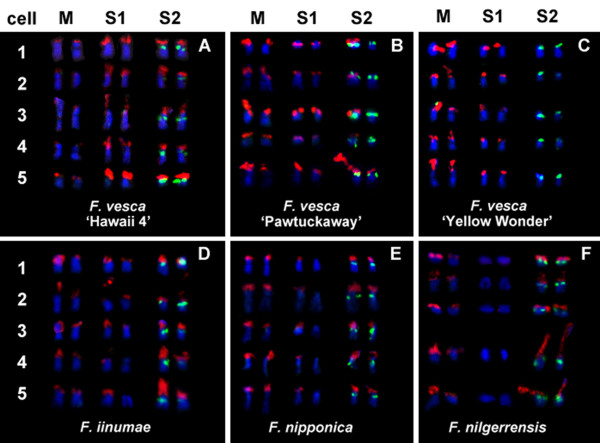
**Variable distribution patterns of 5S (green) and 25S (red) rDNA sites among diploid *Fragaria *genotypes**. Five cells (numbered 1-5) are selected from each of six diploid accessions, which show divergent distribution patterns of rDNA sites. Only chromosomes displaying (or "expected" to display) rDNA FISH signals are shown here (**M: **the medium-sized pair "single-marked" by 25S rDNA signals; **S1: **the small-sized pair "single-marked" by 25S rDNA signals; **S2: **the small-sized pair "double-marked" by both 25S and 5S rDNA signals in the typical pattern). The pattern represented by *F. vesca *'Hawaii 4' here (**A**) is the typical one (6 = 2M+2S1+2S2) shared by most diploid *Fragaria *taxa, while distinctly divergent patterns are observed in very a few accessions shown in **B**. 5 = 2M+2S1+1S2 with an exception as 6 = 2M+2S1+2S2; **C**. 4 = 2M+2S1; **D**. 6 = 2M+2S1+2S2 with an exception as 5 = 2M+1S1+2S2; **E**. 5 = 2M+1S1+2S2; **F**. 4 = 2M+2S2. Within each of most accessions, the distribution pattern among cells is consistent, except for *F. vesca *'Pawtuckaway' and *F. iinumae*. In *F. vesca *'Pawtuckaway' (**B**), the S2 pair displays only one 25S rDNA signal in cells 1 to 4 but two signals in cell 5. In *F. iinumae *(**D**), the S1 pair exhibits two distinct 25S rDNA signals in cells 1 to 3, one distinct and one weak signal in cell 4, and only one signal in cell 5.

Among most diploid accessions examined, a common distribution pattern of rDNA sites was observed, involving three chromosome pairs. In this pattern (Figures 1A-1G, Figure 2A) six 25S rDNA sites were localized on three chromosome pairs, one of which was also marked by a pair of 5S rDNA sites. Among these three chromosome pairs, we designated the medium-sized submetacentric pair that is "single-marked" by 25S rDNA signals as the "M pair". The other two marked pairs (S1 and S2) were the smallest among the seven chromosome pairs, and were either submeta- or subtelo-centric. A pair of 25S rDNA signals was present on one of these two small chromosome pairs, hereafter referred to as the "S1 pair" (for the small-sized, "single-marked" pair), while the small, "double-marked" pair with both 25S and 5S sites was designated the "S2 pair" (Figure 2).

Besides the typical pattern shared by most diploid strawberries, some distinct patterns involving reduced numbers of 25S sites were seen (Figure 2). The S2 pair typically carrying both 5S and 25S rDNA sites lacked the 25S FISH signal on both homologs in all examined cells of *F. vesca *subsp. *vesca *'Yellow Wonder', leaving this accession with only four 25S signals along with two 5S signals (Figures 1K and 2C). Among the diploid accessions, only 'Yellow Wonder' did not have any chromosomes double marked by 25S and 5S rDNA signals (Table 1). In a parallel case, it was the typically single-marked S1 pair that lacked the 25S FISH signal on both homologs (Figures 1H and 2F) in *F. nilgerrensis *(CFRA 1358 and CFRA 1825). Contrastingly, only one homolog of the S1 pair lacked a 25S signal in all examined cells in *F. nipponica *(CFRA 1862, Figures 1I and 2E). Also, although six 25S signals were observed in *F. iinumae *(CFRA 1850, Figure 1G), the signal on one member of the S1 pair was variable among cells, ranging from very small (cells 1 to 3 in Figure 2D) to extremely weak (cell 4 in Figure 2D), and to even invisible (cell 5 in Figure 2D). Finally, in *F. vesca *subsp. *americana *'Pawtuckaway' (CFRA 1948) only one member of the S2 pair had a 25S signal in 24 out of 26 cells (Figures 1J and 2B), while in the other two cells, both S2 homologs had 25S signals (e.g., cell 5 in Figure 2B).

In most diploid accessions, remarkable variability in signal size and intensity was observed among the three 25S rDNA loci as well as between homologs at each 25S locus. (Figures 1 and 2). Yet of the three typically marked chromosome pairs, no one pair had consistently the brightest or least bright 25S signals across the diploid accessions. For instance, in *F. vesca *'Hawaii 4' the 25S rDNA FISH signals on the M pair were remarkably smaller and weaker than the ones on the S1 and S2 pairs, thus showing a "minor M - major S1 - major S2" pattern (Figure 2A); while in *F. nipponica *the S1 pair showed the smallest and weakest 25S rDNA signals, thus presenting a "major M - minor S1 - major S2" pattern (Figure 2E). This variability in allocation patterns of "major" and "minor" 25S rDNA signals were not only observed among different diploid species but also between subspecies (e.g., "minor M - major S1 - major S2" in *F. vesca *subsp. *vesca *'Hawaii 4' versus "major M - major S1 - major S2" in *F. vesca *subsp. *americana *'Pawtuckaway', Figures 2A and 2B), or even between different accessions within a subspecies (e.g., *F. vesca *subsp. *vesca *'Hawaii 4' versus "major M - major S1 - none S2" in *F. vesca *subsp. *vesca *'Yellow Wonder', Figures 2A and 2C). Within a single accession, on the other hand, the allocation patterns were generally consistent among examined cells (Figure 2). Contrastingly, one or two satellites and secondary constrictions, which are cytological markers for transcriptional active rDNA sites, are only sometimes visible in metaphase chromosome preparations in some diploids (Figure 1) and were variously associated with either "major" or "minor" FISH signals.

### Number of 5S and 25S rDNA sites in polyploids

**Figure 3:**
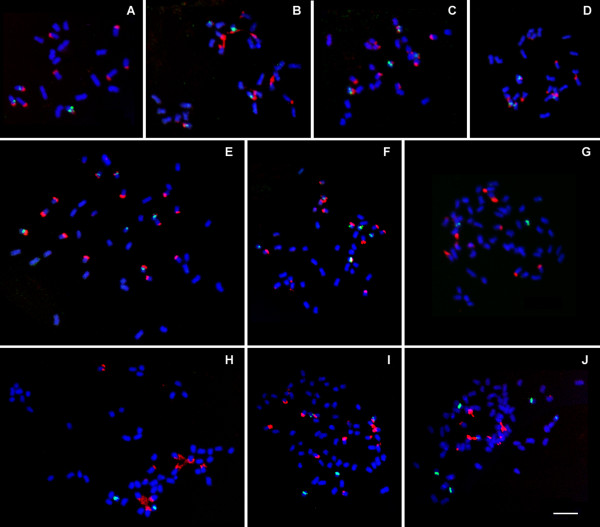
**FISH on polyploid *Fragaria *genotypes with 5S (green signals) and 25S (red signals) rDNA probes**. **A: ***F*. ×*bifera *'GS104' (2*n *= 3*x *= 21); **B: ***F. corymbosa *(CFRA 1911, 2*n *= 4*x *= 28); **C: ***F. gracilis *'GS31' (2*n *= 4*x *= 28); **D: ***F. tibetica *'GS28' (2*n *= 4*x *= 28); **E: ***F. moschata *(CFRA 157, 2*n *= 6*x *= 42); **F: ***F. moschata *(CFRA 376, 2*n *= 6*x *= 42); **G: ***F. chiloensis *subsp. *lucida *(CFRA 1691, 2*n *= 8*x *= 56); **H: ***F. virginiana *subsp. *glauca *(CFRA 1992, 2*n *= 8*x *= 56); **I: ***F. virginiana *subsp. *platypetala *(CFRA 110, 2*n *= 10*x *= 70); **J: ***F. iturupensis *(CFRA 1841, 2*n *= 10*x *= 70). Bar = 5 μm.

*F*. ×*bifera *is a naturally occurring hybrid species and involves both diploid and triploid cytotypes [12]. The accession (GS104) examined in our study was a triploid as shown by chromosome counting. The numbers of 5S and 25S rDNA sites in the triploid *F*. ×*bifera *were three and nine, respectively (Figure 3A). Each of the three 5S rDNA sites was co-localized with a 25 rDNA signal. However, one 5S rDNA signal was obviously larger and stronger than the other two.

The three tetraploid species shared the same numbers of rDNA sites, which were four for 5S and twelve for 25S rDNA (Figures 3B-3D). These numbers are twice the numbers of two 5S and six 25S sites detected in most diploids, and each of the tetraploids' 5S rDNA sites was co-localized with a 25S rDNA site. Although the number and position of rDNA sites was strictly consistent among the tetraploids, differences in signal size and intensity among sites were observed. In *F. gracilis *(Figure 3C) and *F. tibetica *(Figure 3D), 25S rDNA sites among three homologous chromosome groups were not of noticeably different signal intensities, while in *F. corymbosa *(CFRA 1911, Figure 3B), 25S rDNA sites in one "single-marked" group of four chromosomes had larger signals than those of the other two groups. Also, among the total four 5S rDNA sites in *F. corymbosa*, two were larger and stronger than the other two.

In the hexaploid species *F. moschata*, two accessions (CFRA 157 and CFRA 376) were examined. Although eighteen 25S rDNA sites would be expected as a multiple of three times the number seen in most diploids, only seventeen (in CFRA 376, Figure 3F) or sixteen (in CFRA 157, Figure 3E) were detected. Six 5S rDNA sites were found as anticipated in both accessions, but one of them was not co-localized with a 25S rDNA site, and one pair of 5S sites was always much larger and more intensive than the other two pairs in both accessions.

We investigated seven octoploids that represent three subspecies of *F. chiloensis *and three subspecies of *F. virginiana*, and found that each of the octoploid taxa shared common 5S and 25S rDNA site numbers, which were two and ten, respectively (only data for two representative taxa are shown here, in Figures 3G and 3H). These numbers for both 5S and 25S rDNA sites were much less than the anticipated multiples (eight and twenty-four, respectively) of those in most diploids. In addition, no double-marked chromosomes were observed.

A wild genotype (CFRA 110) in *F. virginiana *subsp. *platypetala *was found to be a decaploid by chromosome counting [7]. Our work on the same accession confirmed the ploidy characterization of CFRA 110 by showing 70 chromosomes in a mitotic spread. FISH study detected four 5S and fifteen 25S rDNA sites distributed over 18 chromosomes, of which one was double-marked by a 25S and a 5S rDNA signal (Figure 3I). In *F*. *iturupensis*, the other natural decaploid species found so far in this genus, six 5S and twelve 25S rDNA sites were detected over eighteen chromosomes, and no chromosome was double-marked (Figure 3J). Both decaploids also presented variable signal sizes and intensities among 5S rDNA sites.

## Discussion

Molecular cytogenetic analysis of ribosomal RNA genes by FISH has been performed in many plants, yet studies spanning an extensive polyploid series and encompassing most of the species within a genus are quite rare. In the strawberry genus *Fragaria*, prior to our initial report on *F. vesca *subsp. *vesca *'Hawaii 4' in Shulaev et al. [28], only one unspecified accession of one diploid species, *F. vesca*, had been studied [27]. In the present investigation, nine of the 12 known diploid species as well as several polyploid taxa were studied for genomic distribution of rDNA sites. In total, we extended chromosomal localization of both 5S and 25S rDNA clusters via FISH to 33 accessions representing 25 taxa (species and subspecies), covering ploidy levels from diploid to decaploid.

In all the *Fragaria *species and subspecies examined here, the 5S rDNA signals were displayed in proximal regions of chromosome short arms, while the 25S rDNA signals were on terminal chromosomal regions, indicating that the chromosomal positions of rDNA sites are highly conserved across *Fragaria*. Yet, as detailed below, marked variations in signal intensity were observed among diploid taxa, and intriguing instances and patterns of signal loss were observed, both within and between ploidy levels.

### Typical and exceptional distribution patterns of 5S and 25S rDNA sites among diploid strawberry species

Among and within diploid taxa, the observation of two 5S and six 25S rDNA sites by FISH analysis (Figure 1) was generally consistent with previous findings in *F. vesca *[27,28]. Thus, at the diploid level, one copy of the basic (*x *= 7) *Fragaria *genome is typified by the detectable presence of one 5S locus and three 25S loci, distributed such that one chromosome is "double-marked" by a proximal 5S rDNA locus and a terminal 25S rDNA locus, and two chromosomes are "single-marked" by terminal 25S rDNA loci (Figure 2A). By reasonable inference from its typicality, the described pattern may represent that of the ancestral *Fragaria *genome.

Among diploids, the greatest departures from the typical pattern were two distinct instances of rDNA site loss, each involving a different locus. First, absence of the 25S rDNA signals from the S1 pair left both accessions of *F. nilgerrensis *with only four 25S signals (e.g., CFRA 1358 in Figure 2F). This divergence of *F. nilgerrensis *from the other diploid species contributes to its status as a well-differentiated evolutionary unit, as suggested by the phylogenetic studies of Rousseau-Gueutin et al. [13] and Harrison et al. [30], and also by the sterility of the hybrids resulting from its crosses with other *Fragaria *species [31]. Second, *F. vesca *'Yellow Wonder' also displayed only four 25S signals, but the site losses were from the S2 pair (Figure 2C), also making 'Yellow Wonder' distinct among diploid taxa, including the other studied genotypes of *F. vesca *subsp. *vesca*. Thus, diminution of 25S rDNA site number from six to four has occurred at least twice among diploid *Fragaria*.

Polymorphisms were also detected in size and intensity of 25S rDNA FISH signals, which we described as "major" (large and strong) and "minor" (small and weak) signals, between different loci in the various diploid taxa. Yet of the three typically marked chromosome pairs, no one pair had consistently the brightest or least bright 25S signals across the diploid genotypes. Thus, in contrast to the conservation of a typical pattern of rDNA site distribution, the allocation pattern of 25S rDNA signal intensities among chromosome pairs varied among diploid genotypes to an extent that precluded typification. Furthermore, polymorphism in signal intensity and/or presence versus absence was observed between homologous 25S rDNA sites in many diploid genotypes. Signal absences from one but not both members of a typically marked pair occurred in three cases: *F. nipponica *(Figure 2E), *F. iinumae *(Figure 2D), and a genotype ('Pawtuckaway') of *F. vesca *subsp. *americana *(Figure 2B). The latter pattern of variability between homologs was consistently seen on separately prepared slides suggesting that this variability was not due to experimental artifact. Based upon available data, it would be premature to speculate about the possibility that unbalanced signal intensity between homologs is a precursor to eventual locus loss, culminating in a diminution of 25S site number from the typical six to four. However, all the variations summarized above indicate that the 25S rDNA arrays in diploid *Fragaria *exist in a dynamic state.

### Characterization of 25S rDNA arrays in diploid strawberries: conservation of site number and chromosomal position vs. dynamics of copy number

As FISH is considered to be a semi-quantitative technique [32], it is reasonable to expect that size and intensity of hybridization signals is an indicator of targeted sequence copy number. Thus, the polymorphism of 25S rDNA signal intensities revealed among diploid *Fragaria *may imply different repeat copy numbers among different rDNA sites. Loss of 25S rDNA signal(s) could be due to a complete elimination of entire sites, or perhaps only to the loss of most copies of 25S rDNA repeats at the respective site(s), resulting in a diminished signal or leaving too few repeats to be detected by FISH. Such outcomes could be attributable to spontaneous deletion of an rDNA-containing fragment from the short arm of the chromosome(s) [33], or to unequal crossing over, which could lead to a loss (and/or gain) of repeats from different sites [34].

Any rDNA site is a stretch of DNA with sequence homology to other rDNA sites on other chromosomes [35]. Thus, physical association of rDNA clusters between both homologous and nonhomologous sites is possible, supported by the fact that rDNA-bearing chromosomes appear to be non-randomly associated with each other at mitotic metaphase [36]. It is widely accepted that association of genes with highly repetitive sequences would increase the opportunity for unequal exchanges. The distal chromosomal position of rDNA sites in strawberry may facilitate this association, which could lead to unequal exchanges and rDNA repeat duplications/deletions, and therefore changes (both increases and decreases) in copy number between both homologous and nonhomologous sites. Size polymorphism of the hybridization signals, among and within homologous sites could be explained by such events. On the other hand, somatic exchanges taking place between different sites could make homogenization and changes in rDNA copy number occur especially quickly [36]. Therefore, it is reasonable to suggest that the rDNA repeats in strawberry are in a highly dynamic state because of their terminal positions and potentially high degree of association between sites. However, the high conservation in rDNA site number and chromosomal location, despite their apparent high dynamics of copy number among sites, may indicate conserved genome organization among strawberry species, at least for chromosomal segments involving rDNA sites.

When more than one pair of 18S-25S rDNA sites is present in the genome, some sites may be inactive [34]. When sites are active, a secondary constriction is typically visible. Once genes are inactivated (silenced), the constriction disappears, even though the sequence is still present and detectable by FISH [37]. In strawberry, satellites have been reported on a small pair of chromosomes in five diploid species in previous karyotype studies [23,24]. In our work, one or two satellites and secondary constrictions are sometimes visible in metaphase chromosome preparations in some diploids (Figure 1), and were always associated with a 25S rDNA FISH signal, irrespective of signal size. In reports on some plants, active rDNA clusters that can form nucleolar organization regions (NORs) and produce large and intensive FISH signals have been described as major loci, while the ones without transcription activity but are still detectable by FISH as weak signals are described as minor loci (e.g., *Hordeum*) [38]. So far, whether all or only some of the rDNA sites in strawberry are actively transcribed and form NORs awaits resolution by further work. The terms "major/minor sites" applied in the Results section are not intended to refer to the transcriptional activity of rDNA sites, but only to relative signal brightness.

### Proportional increase of rDNA site number in lower (3*x* - 6*x*) polyploid strawberries

The triploid cytotype of the hybrid *F. ×bifera *was previously inferred to possess one copy of the *F. vesca *genome and two copies of the *F. viridis *genome [12]. Three 5S and nine 25S rDNA sites observed in this triploid cytotype constitute simple multiples of both rDNA site numbers in its two diploid progenitors. The one larger and stronger 5S rDNA site is probably from *F. vesca*, while the two smaller ones are from *F. viridis*, in consideration of the copy number of subgenomes provided by the two donors.

In three tetraploids, multiples of 5S and 25S rDNA site numbers (Figure 3B-3D) are increased in proportion to the increase in whole genome copy number (i.e., in comparison to the typical diploids, the tetraploids had twice as many chromosomes and twice as many detected 5S and 25S rDNA sites). To date, the alternate possibilities of auto- or allo- polyploidy origin have not been resolved in these tetraploids [13]. Various diploid species occurring in respectively overlapping geographical areas have been proposed as putative ancestors. Due to the highly conserved genomic distribution pattern of rDNA sites among diploids, no specific species were identifiable as putative ancestors of the tetraploids based on rDNA-FISH data. However, variable allocation of 25S rDNA signal intensities among loci between these tetraploids at least implies that the diploid ancestry of *F. corymbosa *may be distinct from those of either *F*. *gracilis *or *F. tibetica*. Lundberg et al. [39] suggested that *F. corymbosa *was an allotetraploid. The size polymorphism of 5S rDNA signals in *F. corymbosa *observed in our work is possibly supportive to this hypothesis.

In the hexaploid species *F. moschata*, six 5S and eighteen 25S rDNA sites would be expected as three times those in most diploids. In fact, six 5S rDNA sites appeared as distinct FISH signals, but one or two fewer signals for 25S rDNA sites were shown in, respectively, *F*. *moschata *genotypes CFRA 376 and CFRA 157 (Figure 3E and 3F). *F. moschata *was shown to be an allopolyploid and its subgenome donors include *F. vesca *and *F. viridis*, which were suggested by DNA molecular studies [13,39,40]. In *F. vesca*, some genotypes (e.g., 'Yellow Wonder' and 'Pawtuckaway') have fewer 25S rDNA sites than six. Thus, the 25S rDNA site number less than eighteen in a hexaploid would not be surprising, if any *F. vesca *genotype(s) having less than six 25S rDNA sites were involved in its origin. Alternatively, one or two rDNA sites could have been diminished or lost by loss of most or all of its repeats during or after the arising of the hexaploid. When Rousseau-Gueutin et al. [13] studied two low-copy gene sequences for the construction of phylogenetic trees of *Fragaria *species, they linked *F*. *moschata *to both *F. vesca *and *F. viridis *in the tree based on the sequence analysis of the gene *GBSSI-2*, but detected no affinity between *F. moschata *and *F. viridis *in the tree from the *DHAR *gene, for which physical elimination of one homoeologous copy of this gene was proposed to be a possible reason.

In genotype CFRA 157, five of the six 5S rDNA sites are co-localized with 25S rDNA sites, which means loss of one 25S rDNA site (if the latter hypothesis discussed above is true) has occurred on one of the six "double-marked" chromosomes and the second one on a "single-marked" chromosome. These two chromosomes could not be homologs, suggesting that elimination of 25S rDNA sites could occur simultaneously on non-homologous chromosomes. Therefore, in species with a high site number, loss of rDNA copies could proceed very quickly simply because there are many opportunities for occurrence of this event. This speculation gains support from the cases of octo- and deca-ploid strawberries, in which eight 5S and twenty-four 25S rDNA sites, and ten 5S and thirty 25S rDNA sites, respectively, would be expected but much less are actually observed.

### Remarkable rDNA site number reduction in octoploid strawberries

Each of the two wild octoploid species includes multiple subspecies. In this work, we examined three subspecies of *F. chiloensis *and of *F. virginiana*, respectively. Numbers of 5S and 25S rDNA sites are consistent among all these subspecies, and a strong reduction in rDNA site number for both kinds of rDNA was observed as compared with proportionate multiples of the typical diploid numbers. The strong reduction in rDNA site number might be attributable to two factors. First, more than a half of the rDNA sites inherited from the lower-ploidy ancestors might have failed to participate in associations occurring among other rDNA-bearing chromosomes, where homogenization through unequal crossing-over and gene conversion could have maintained homology of their DNA sequences and therefore their transcriptional function. Thus, susceptibility to loss of some rDNA arrays may be due simply to the high initial number of rDNA sites. Though rDNA site number is expected to be correlated with genome size, perhaps only a restricted number of rDNA sites under a certain threshold could be associated together and be maintained in homology, and this low number might be sufficient to support normal cellular activity. Thus, extra sites beyond that restricted or necessary number would suffer accumulation of mutations from lack of homogenization forces, and be subject to elimination.

A second possibility is nucleolar dominance, as originally described by Navashin [41] in some species of *Crepis*. This phenomenon occasionally occurs in natural allopolyploids as well as synthetic interspecific hybrids [42], which show exclusive transcriptional activity of genes encoding 18S, 5.8S, and 25S rRNA that belong to one of the parental genomes with concurrent lack of expression of rDNA from the other parental genome [43]. However, how fast one of the parental rDNA repeat types may be removed from the hybrid genome and by what evolutionary forces is currently unresolved. If nucleolar dominance also occurs in strawberry, loss of rDNA sites could be explained as occurring after loss of the transcriptional activity of these sites.

As an explanation for rDNA site loss, the hypothesis based on nucleolar dominance could be integrated with that of high initial copy number if association of rDNA sites is demonstrated to occur only between those derived from a subset of the diploid ancestors. Although genome composition of octoploid strawberries has not been fully elucidated [44], putative subgenome donors including ancestors of *F. vesca *and *F. iinumae *have been strongly supported by phylogenetic analysis on DNA sequence data of multiple nuclear genes [1,13], whereas in the phylogenetic tree constructed on nuclear ITS sequences [45], *F. iinumae *is not clustered with any octoploids but is a sister species to all the others. As nuclear ITS is included in the 45S rDNA unit, it is reasonable to speculate that rDNA site loss could be subgenome-specific and that rDNA repeats from the ancestral *F. iinumae *subgenome(s) were lost during or after the establishment of the ancestral octoploid(s).

Davis et al. [46] noticed the evident diminution of genome size by 12% to 16% in two octoploid cultivars as compared with an expectation of four times the size of a diploid genome. This diminished size could be due to events, such as losses of DNA segments, that occurred during or after the origination of the octoploids [46]. The marked loss of rDNA sites in octoploids could have been a part of a broader, generalized loss of DNA segments, or could have occurred via a separate and distinct mechanism.

### Origins of higher ploidy strawberries

Identical site numbers of both 5S and 25S rDNA among all the octoploid subspecies of *F*. *chiloensis *and *F. virginiana *suggest that the wild octoploid species are closely related and very likely share a common genome composition or even have a common ancestor, as proposed by phylogenetic analysis based on different DNA sequences [13,30,45]. Moreover, conservation of 5S and 25S rDNA site numbers among the octoploid species and subspecies suggests that site loss to observed levels may have been an early event, preceding the divergence of the various octoploid taxa from a common octoploid ancestor. The origin of *F*. *moschata *may have followed an independent and perhaps more recent evolutionary pathway as compared with the octoploid lineage(s), given that *F. moschata *still "maintains" most of its rDNA sites.

The presence of four 5S and fifteen 25S rDNA sites suggests that the decaploid cytotype for CFRA 110 of the *F*. *virginiana *subsp. *platypetala *originated from doubling of an interspecific hybrid between an octoploid and a diploid species, which are probably *F. virginiana *and an American subspecies of *F. vesca*, respectively, due to the North American geographic collection site of CFRA 110. Thus, this decaploid cytotype could be comprised of two 5S and five 25S rDNA sites from the *F. vesca *progenitor, and two 5S and ten 25S rDNA sites from the octoploid progenitor. Contrastingly, the site numbers of six for 5S and twelve for 25S rDNA in decaploid *F. iturupensis *do not fit an origination model involving a simple combination of an octoploid and a diploid. In such an origin, the decaploid would be expected to have fewer (four) 5S sites and more (15 or 16) 25S sites, as seen in decaploid CFRA 110. Instead, the rDNA site numbers in *F. iturupensis *imply a different and probably more complex origin of this decaploid species as compared with CFRA 110.

### Identification of individual chromosomes by rDNA markers

Due to the extremely small size (i.e., 0.61-1.85 μm for strawberry) [25] and morphologically minimal differentiation of *Fragaria *chromosomes, chromosome-specific markers provided by FISH are needed for the identification of individual chromosomes, and are critical for tracking homo- or homoeo-logous chromosomes among species and in polyploids.

A previous rDNA-FISH technique performed on *F. vesca *enabled the construction of a karyotype with three pairs of marked chromosomes in diploid strawberry [27]. Confirmatory results were obtained in our lab recently on *F. vesca *'Hawaii 4' [28]. For most accessions of *F*. *vesca*, our data presented here are congruent with the two previous studies. Among the three pairs of chromosomes with rDNA markers, one pair is double marked by 25S and 5S rDNA, and the other two single marked pairs can be distinguished by their different size and/or signal intensities of 25S sites they bear. The other eight chromosomes in a diploid complement are still challenging for even matching of homologs, except for the largest pair, which can be grouped by its size in most cells. To date, various genomic resources for *F. vesca *including a fosmid library [47], mapped and annotated fosmid clones [10], a BAC library [48], and the draft 'Hawaii 4' genome [28] have been established. Assembly has been anchored to the genetic linkage map into seven pseudochromosomes. These all give good opportunities for developing new, chromosome-specific probes that can expand the scope of karyotypic resolution in *Fragaria*.

We established several fosmid clones for identification of more individual chromosomes besides the rDNA marked ones in *F. vesca *(unpublished data). A comprehensive molecular karyotype in strawberry could be constructed. By finding sequence homology between 25S and 5S rDNA probes used for chromosome identification and scaffolds mapped to the pseudochromosomes of the 'Hawaii 4' linkage map, the chromosome pair double marked by 25S and 5S rDNA sites and the other small chromosome pair single marked by 25S rDNA signals correspond to pseudochromosome VII and pseudochromosome VI, respectively [28]. As additional probes are developed, the seven linkage groups or pseudochromosomes will be assigned to each specific chromosome so that any sequences of interest defined in a certain pseudochromosome could be verified cytologically along real chromosomes with the assistance of chromosome-specific landmarks in the comprehensive molecular karyotype.

### rDNA site numbers in the Rosaceae family

Physical mapping of rDNA sites by FISH has been performed in several genera of the Rosaceae family, but very few species have been examined within each of these genera. In overview, the number of detected 25S rDNA sites in diploid rosaceous species has been either two (in *Rosa *and *Rubus parvifolius*) [49-52], four [49], or six (in *Prunus*) [53-55]. Apple (*Malus *x *domestica*), in which a relatively recent genome-wide duplication is indicated [56], has a distinctively high chromosome number of 2*n *= 34 in Rosaceae, and eight 25S rDNA sites were observed in it [57]. For 5S rDNA, a constant number of two for diploids has been observed in most of these genera except for *Prunus *[53,54] and *Rosa *[50,52], in which four sites have been found. In terms of chromosome position, all the rosaceous species including *Fragaria *exhibit a similar distribution pattern, in which 25S rDNA repeats are clustered at terminal regions while 5S rDNA sites are in interstitial and proximal regions of chromosomes. However, "double-marking" by both 5S and 25S rDNA as seen in *Fragaria *was only reported for diploid *Prunus subhirtella *[54] and a pentaploid *Rosa canina *[50]. Comparative molecular cytogenetic analysis in the Rosaceae will benefit from the development of additional probes targeting conserved sequence sites at multiple chromosomal locations.

## Conclusions

This report describes the first molecular cytological study of comparative genome organization in *Fragaria*, and reveals the extent to which the genomic distribution of rDNA sites is conserved among and within species and ploidy levels, as well as dynamics of 25S rDNA repeats, in this economically important genus. Per basic genome copy, one proximal 5S and three terminal 25S rDNA loci were largely but not uniformly conserved in diploids (2*x*) and lower polyploids (3*x*, 4*x *and 6*x*), but a marked reduction in site number was seen in higher polyploids (8*x *and 10*x*). Based upon shared genomic distribution patterns of rDNA sites, a common origin of the *Fragaria *octoploids is suggested; however, the distinctly different patterns seen in the two recently identified decaploids suggest that these originated independently. In the Rosaceae family, *Fragaria *was the first genus in which a systematic molecular cytogenetic study has been done, thereby providing a comparator for genomic studies in other rosaceous species that could, for instance, reveal common or differing trends in rDNA locus evolution following polyploidy.

## Methods

### Plant material

Twenty-five taxa (species and subspecies) belonging to *Fragaria *were sampled (Table 1). These included 12 diploid taxa, a triploid cytotype of *F*. ×*bifera*, three tetraploid species, hexaploid *F. moschata*, seven octoploid taxa, and two decaploids. In total, 33 genotypes belonging to 17 *Fragaria *species were examined. The geographic distributions of *Fragaria *are described in Folta and Davis [1] and Hummer et al. [58].

### DNA isolation and probe preparation

Total genomic DNA was isolated from 0.1 g unexpanded leaf tissue using a modified CTAB protocol [59]. The primers for PCR amplification of 25S rDNA were designed based on 25S rDNA from *Arabidopsis thaliana *[60], and their sequences were: 25SF - 5'ACGGACCAAGGAGTCTGACATG; and 25SR - 5'CGCTTTCACGGTTCGTATTCG. Using genomic DNA of *F. vesca *'Yellow Wonder' as a template, PCR was performed with an initial denaturation at 94°C for 3 min, followed by 30 cycles of 94°C for 30 sec, 55°C for 20 sec, and 72°C for 2 min, followed by a 10-min 72°C final extension. Products were purified by ethanol precipitation and labeled by a nick-translation reaction using biotin-16-dUTP (Roche Diagnostics, Indianapolis, Indiana). The 5S rDNA primers were as in Brown and Carlson [61] with modifications based on the 5S rDNA sequence from strawberry of *F. vesca *'Hawaii 4' [28], and their sequences were: 5SP1 - 5'GAGGGATGCAACACGAGGCC; and 5SP2 - 5'CGGATGCGATCATACCAGCA. The labeling reaction was performed by PCR using template DNA from *F. vesca *'Yellow Wonder', and dNTPs mixed with DIG-11-dUTP (Roche Diagnostics, Indianapolis, Indiana). The reaction was initially denatured at 94°C for 3 min, then subjected to 30 cycles of 94°C for 30 sec, 50°C for 20 sec, and 72°C for 1 min, followed by a 10-min 72°C final extension.

### Chromosome preparation and Fluorescence *in situ* hybridization

Pretreatment of strawberry root tips was performed as described by Nathewet et al. [25]. Then the root tips were rinsed briefly in 0.075 M KCl and fixed in 3:1 methanol:acetic acid at 4°C for at least 24 hrs. Fixed root tips were digested in 2% Cellulase 'Onozuka' RS (Yakult Honsha, Tokyo, Japan) and 0.05% Macerozyme R-10 (Yakult Honsha, Tokyo, Japan) at 37°C for 20 min and transferred to 0.075 M KCl for 10 min. Then the root tips were fixed in 3:1 methanol:acetic acid at 4°C. Chromosome spreads were made by the smearing method as described by Liu et al. [18], and slides were stored at -80°C until FISH analysis. FISH experiments were carried out with some modifications of the procedure of Liu et al. [62]. Briefly, slides were pretreated using RNase (100 ng/ml in 2xSSC) and pepsin (0.01% in 10 mM HCl), then denatured in 70% formamide for 3 min at 80°C. 25S and 5S rDNA probes in 2xSSC, 50% deionized formamide, and 10% dextran sulphate were denatured for 8 min at 90°C, then applied to the denatured slides and hybridized overnight at 37°C. After post-hybridization washes, signals were detected using streptavidin-Cy3 (Sigma) and anti-DIG-FITC (Roche Diagnostics, Indianapolis, Indiana). Slides were mounted and counterstained in Vectashield (Vector Laboratories) containing 2 μg/ml 4',6-diamidino-2-phenylindole (DAPI). Photographs were taken with a ZEISS Axioplan 2 Imaging fluorescence microscope equipped with AxioCam MRm CCD camera (Carl Zeiss, Jena, Germany) and AxioVision 4.8.1 software (Carl Zeiss, Jena, Germany). The images were analyzed with Adobe^® ^Photoshop^® ^CS3 and treated for color contrast and uniform brightness only. At least 5 mitotic metaphase complements per accession were scored.

## Authors' contributions

BL designed and performed the research, and drafted the manuscript. TMD conceived of the study and helped to draft the manuscript. Both authors read and approved the final manuscript.

## Authors' information

Both authors are at Department of Biological Sciences, University of New Hampshire, Durham, NH 03824, USA.
